# Editorial: Pharmaceutical insights into the triazoles: Recent advances

**DOI:** 10.3389/fchem.2023.1149133

**Published:** 2023-02-01

**Authors:** Xianqing Deng, Haopeng Sun, Bahaa G. M. Youssif

**Affiliations:** ^1^ Health Science Center, Jinggangshan University, Ji’an, China; ^2^ School of Pharmacy, China Pharmaceutical University, Nanjing, China; ^3^ Department of Pharmaceutical Organic Chemistry, Faculty of Pharmacy, Assiut University, Asyut, Egypt

**Keywords:** triazole, synthesis, anticonvulsant, anticancer, click chemistry

Triazole refers to a heterocyclic with molecular formula C2H3N3, having a five-membered ring of two carbon and three nitrogen atoms. It had reported that compounds containing triazole exhibited broad biological activities, such as antimicrobial, analgesic, anti-inflammatory, anticonvulsant, antineoplastic, antimalarial, antiviral, antiproliferative, and anticancer activities. The electron richness and aromaticity of triazole enable it to freely bind with a wide range of biomacromolecules by interactions of pi–pi bonds, H bonds, and ion-dipole bonds. In recent years, the number of research articles on triazole use in medicinal chemistry has increased substantially. Triazoles are becoming potential drug candidates for the scientific community. Regarding the Research Topic, the synthesis method and pharmacological effects of triazole derivatives are very attractive and prospective.

Dai et al. summarized the synthetic methods of 1,2,3-/1,2,4-triazoles combined with the progress of triazole over the past 2 decades. Several main synthetic methods from various nitrogen sources were summarized. The synthetic methods were sorted according to the key starting material utilized for the production of the 1,2,3-/1,2,4-triazole backbones, and some new synthetic methods to access triazole derivatives with biological activities were presented.

Song et al. discovered a skeleton of triazolopyrimidine for the development of new antiepileptics (AEDs). The design, synthesis, and *in vivo* anticonvulsant activity evaluation of triazolopyrimidines, and pyrazolopyrimidines were reported. Most of the triazolopyrimidines showed anticonvulsive activity in the maximal electroshock (MES) and pentetrazol (PTZ)-induced seizure models. The compound **6d**, holding a median effective dose (ED50) of 15.8 and 14.1 mg/kg against MES and PTZ-induced seizures, respectively, was found to be the most potent one. The protection index (PI) value of **6d** was significantly higher than that of some available AEDs. Moreover, the experimental investigation of compound **6d**’s mechanism of action suggested that compound **6d** works as an anticonvulsant agent by regulating the GABA function.

Ullah et al. summarized backbone and/or functionalized fluorinated 1,2,3- and 1,2,4-triazoles with anticancer, antibacterial, antifungal, antiviral, antimicrobial, herbicidal, inhibitory, antioxidant, antagonistic, antimalarial, and anti-inflammatory properties. It is revealed that many biologically and pharmacologically active fluorine-bearing triazoles have been extensively synthesized by the facile click chemistry approach. Many promising and valuable fluorine-incorporating triazoles’ analogs were reviewed as potential lead candidates for antiviral and anticancer agents. It is conclusion that the development of fluorine-incorporating triazoles toward clinical applications is promising.

Mortazavi et al. reported the MET inhibitory effects of 10 novel quinazolinone hydrazine triazole derivatives. The effective activity of CM9 and CM10 derivatives against cancer cells was investigated and confirmed. As the most promising one, CM9 inhibited MET and FLT4 (VEGFR3) kinase activity with an IC50 value of 22.76 and 5.01 µM, respectively. Eventually, important structural features for the interactions of CM9 with MET and FLT4 (VEGFR3) kinases were verified by Molecular docking and molecular dynamics simulation studies.

This Research Topic shows the importance and versatility of the triazole skeleton in organic and medicinal chemistry applications. Explore and obtain highly pharmacologically active compounds with antiepileptic and anticancer properties for the search and development of potential drug candidates and scaffolds. This Research Topic aims to highlight the state-of-the-art achievements of triazole skeletons in drugs and medicinal chemistry to inspire and guide future directions in the field.

